# Entrapment of a laryngotracheal topical anesthesia kit during tracheobronchial foreign body removal: a case report

**DOI:** 10.1186/s12871-018-0529-1

**Published:** 2018-06-01

**Authors:** Xi-Yang Zhang, Yun Han, Ya-Bing Zhang, Ke-Xuan Liu, Bin Liu

**Affiliations:** 1 Department of Anesthesiology, Nan Fang Hospital, Southern Medical University, Guangzhou, Guangdong China; 2 The First Clinical Medical College, Southern Medical University, Guangzhou, China; 3 Department of Anesthesiology, West China Hospital of Sichuan University, Chengdu, China

**Keywords:** Child, Foreign body, Anesthesia, Airway

## Abstract

**Background:**

In order to reduce the irritation of the airway during tracheobronchial foreign body (TFB) removal, tracheal surface anesthesia is usually performed using a laryngotracheal topical anesthesia (LTA) kit (LTA20, Highgreen Medical Technology Company, China), but difficulty in withdrawing the LTA kit is rarely reported. We present a case of a difficulty to withdraw the LTA kit due to its entrapment by the movement of a TFB.

**Case presentation:**

A 1-year-old girl was undergoing TFB removal. After the surgeon completed the tracheal surface anesthesia, the girl suddenly suffered from bucking, leading to the dislodgment of the TFB to the subglottic region, complicating the withdrawal of the LTA applicator. At the same time, the girl’s oxygen saturation (SpO_2_) decreased to 91% and her heart rate dropped from 150 to 100 bpm. Atropine and succinylcholine were administered intravenously immediately, then the surgeon tried to free the TFB by pushing it back into the trachea, after which the LTA applicator was easily withdrawn, and TFB was removed successfully. The girl was discharged from hospital without any complications 2 days later.

**Conclusion:**

This case report draws our attention to a significant anesthetic clinical consideration during the application of topical anesthesia on the trachea for TFB removal. The possibility of coughing or bucking can lead to migration of the TFB with subsequent airway obstruction, so the depth of anesthesia must be sufficient to prevent harmful reflexes. Also, strong teamwork and good communication are paramount to avoid serious complications.

## Background

TFB aspiration is a common life-threatening accident in children, especially those under age 4 [1]. Rigid tracheobronchoscopy is considered the gold standard technique for the management of TFB removal. Because of immature protective mechanisms and relatively narrow airways, severe complications, such as desaturation, laryngeal edema, bronchospasm, pneumothorax, pneumomediastinum, tracheal or bronchial laceration, and even cardiac arrest, can occur with attempting at TFB removal [2]. LTA applicator (LTA20, Highgreen Medical Technology Company, China) is usually used to perform tracheal surface anesthesia, so as to relieve the likelihood of a harmful reflex induced by rigid tracheobronchoscopy. To date, however, difficulties in withdrawing a LTA applicator due to its entrapment by movement of a TFB, with the potential to lead to serious complications, has not been reported.

## Case presentation

The patient’s father provided written consent to the use the patient’s medical information for research and publication.

**Fig. 1:**
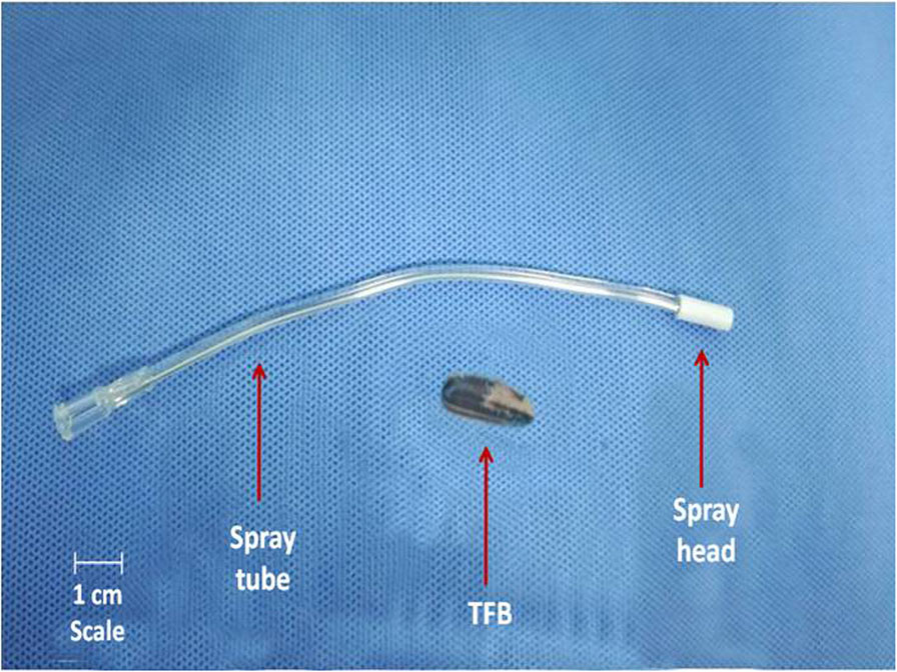
Laryngotracheal topical anesthesia (LTA) kit and tracheobronchial foreign body (TFB)

A 1-year-old girl (weight 10 kg) had suffered from a cough with sputum production for more than 2 days. She had a medical history of having swallowed a TFB 2 days earlier. The physical examination was normal, except for a wheezing sound in the right lung. A chest computed tomography scan revealed an 8 × 4 × 21 mm^3^ mass in the trachea near the carina, which given the patient’s history, was suggestive of the diagnosis of a TFB aspiration. After careful preoperative preparation, we scheduled the patient to undergo an emergency rigid tracheobronchoscopy to remove the TFB under general anesthesia. In order to keep the patient in spontaneous breathing, the combination of intravenous and inhalant anesthesia with propofol, fentanyl, and sevoflurane was planned to be administered. In the operating room, standard monitoring was installed, including SpO_2_, noninvasive blood pressure, and an electrocardiogram. Before administration of anesthesia, the child was premedicated with atropine (0.1 mg) and dexamethasone (2 mg)intravenously. Then anesthesia induction was performed with 8% sevoflurane carried by 6 L/min oxygen flow. After the patient became unconscious, anesthesia was maintained with 3–5% sevoflurane and 1 L/min oxygen flow for more than 5 min. Before rigid tracheobronchoscopy introduced into the trachea, the child received propofol (20 mg) and fentanyl (10 μg) intravenously to deepen the anesthesia. After 1–2 min, when her lower jaw was flabby, the surgeon introduced a LTA applicator into the trachea under the guidance of rigid tracheobronchoscopy, and then sprayed topical 1% lidocaine on the surfaces of the vocal cords and trachea. After the surgeon applied the topical anesthetic to the trachea, the girl suddenly suffered from bucking, which made it difficult to withdraw the LTA applicator**.** The surgeon quickly examined the opening of the main trachea using rigid tracheobronchoscopy, and found the TFB had migrated to the subglottic region against the LTA applicator. In this situation, manual ventilation became impossible and within half a minute, the patient’s SpO_2_ decreased to 91% and her heart rate dropped from 150 to 100 bpm. We administered an intravenous injection of atropine (0.3 mg) and succinylcholine (10 mg). Meanwhile, the surgeon tried to free the TFB by pushing it back into the trachea, after which the LTA kit was easily withdrawn. We then mask-ventilated the lungs successfully. The patient’s SpO_2_ quickly increased to 97% and her heart rate rose to 140 bpm. After deepening the anesthesia with propofol (20 mg) intravenously, the surgeon successfully grabbed and removed the TFB under jet ventilation (Fig. 1). Afterwards, the girl’s condition improved quickly and she recovered uneventfully. She was discharged from the hospital 2 days later without complications.

## Discussion and conclusions

There are 2 primary ventilatory models for guiding anesthetic management during TFB removal: spontaneous respiration and controlled ventilation [3]. Generally, the use of spontaneous respiration without a muscle relaxant provides continuous ventilation, and may not result in complete airway obstruction. However, when using this technique, the anesthetist is challenged to maintain the anesthetic depth, and they usually add topical anesthesia to suppress airway reflexes. The use of controlled ventilation with a muscle relaxant may facilitate the avoidance of patient coughing and bucking, and facilitate the extraction of a TFB, but this technique has some potential risks, such as barotrauma and dislodgment of the foreign body, especially when using jet ventilation through a thin catheter.

The retrospective study of Chai et al. suggests that applying topical lidocaine to the oropharynx, glottic structures, or the subglottic region is helpful and reduces the amount of anesthetics needed to remove a TFB [4], but existing data on the effectiveness of this technique remains mixed [5]. Although our hospital commonly uses a LTA kit to carry out tracheal surface anesthesia, the use of topical lidocaine anesthesia may sometimes result in adverse events, such as bucking, breath holding, laryngospasm, and body movement. We report a rare, but potentially serious incident of an LTA applicator becoming entrapped by a TFB.

In our case, the LTA kit together with the TFB almost completely blocked the subglottic region, leading to a potentially life-threatening situation. We speculate that the cause for this obstruction was inadequate anesthesia depth. We used general anesthesia with spontaneous respiration, so the anesthesia was not deep enough to prevent harmful reflexes to the topical lidocaine spray, such as bucking and body movement. These reflexes resulted in the movement of the TFB and airway obstruction.

The complication rates associated with controlled ventilation vs. spontaneous respiration are similar, except for lower incidence of laryngospasm when controlled ventilation is performed [3]. In our case, the surgeon was able to extract the LTA kit after the administration of succinylcholine; nevertheless, attention should be paid to the possibility of complete airway obstruction when surgeons prepare for such procedure. In another reported case, a foreign body was lodged in the subglottic region, but unlike us, the surgeon successfully removed the seed in pieces without using succinylcholine [6].

As reported, strong teamwork, especially good communication between the medical care providers, is vital to the improvement of patient outcome, the prevention of potentially avoidable complications, and thus the reduction of morbidity and mortality [7]. Therefore, as for the enhanced intraoperative management in our case, we thought that frequent communication between the anesthesiologist and the surgeon throughout the case was extremely important, as insertion of rigid tracheobronchoscopy, spraying topical 1% lidocaine, clamping of the TFB, and other stimulation required to maintain sufficient anesthetic depth.

In a word, we present a case of a difficulty withdrawing a LTA applicator during attempted TFB removal. This case report highlights an important clinical anesthetic consideration when applying topical anesthesia on the trachea for TFB removal. Anesthesia providers should be prepared for the possibility of coughing and bucking, leading to migration of the TFB with subsequent airway obstruction. This situation may possibly be avoided by ensuring an adequate depth of anesthesia prior to the application of lidocaine to the trachea that relieves airway reflexes. Also, facing the event of airway obstruction, preparedness and team communication are paramount to avoid serious complications.

## Abbreviations

LTA: Laryngotracheal topical anesthesia
SpO_2_: Oxygen saturation
TFB: Tracheobronchial foreign body
